# Pyrosequence Read Length of 16S rRNA Gene Affects Phylogenetic Assignment of Plant-associated Bacteria

**DOI:** 10.1264/jsme2.ME11258

**Published:** 2012-02-22

**Authors:** Takashi Okubo, Seishi Ikeda, Akifumi Yamashita, Kimihiro Terasawa, Kiwamu Minamisawa

**Affiliations:** 1 Graduate School of Life Sciences, Tohoku University, Katahira, Aoba-ku, Sendai 980–8577, Japan; 2 Memuro Research Station, National Agricultural Research Center for Hokkaido Region, Shinsei, Memuro-cho, Kasai-gun, Hokkaido, 082–0081, Japan

**Keywords:** Bacterial community, phylogenetic analysis, 16S rRNA gene, plant-associated bacteria

## Abstract

Pyrosequence targeting of the 16S rRNA gene has been adopted for microbial communities associated with field-grown plants. To examine phylogenetic drifts according to read length and bioinformatic tools, original and chopped sequences (250–570 bp) covering the V1–V4 regions of 16S rRNA genes were compared using pyrosequence and Sanger reads of rice root microbiomes. The phylogenetic assignment at genus level depended on read length, especially in the genus *Bradyrhizobium*, which is one of the ecologically important bacterial genera associated with plants. We discuss the methodology of phylogenetic assignments of plant-associated bacteria by 16S rRNA pyrosequence.

Diverse microorganisms live in and on plants. Microbial communities of field-grown plants have been surveyed by the use of 16S rRNA sequence-based methods in many studies. In most cases, clone libraries were constructed by PCR amplification of 16S rRNA gene segments (7, 13, 14).

The GS-FLX Titanium pyrosequencer (454 Life Sciences, Branford, CT, USA) has now been used to analyze plant-associated bacterial communities (Ikeda *et al.* unpublished). Pyrosequencing yields more than half a million sequences per run, and enables analysis of many more samples simultaneously at considerably lower cost; however, its limitations are short and variable read lengths, and a lower sequence quality than those of Sanger dideoxy sequencing (5). Generally, for 400-base partial 16S rRNA sequences, the Ribosomal Database Project (RDP: http://rdp.cme.msu.edu/) Classifier tool is accurate down to genus level (19); however, 400-base partial 16S rRNA sequences generated by pyrosequencers might not be accurately classified at genus level in some taxa because of the sequencers’ higher error rates (10). Thus, comparative studies are needed to assess the effects of different primer sets and sequencers.

The effect of read length on the phylogenetic analysis of 16S rRNA gene sequences of rice-root-associated bacteria was examined using samples constructed from 454 and Sanger reads to simulate different read lengths (Table 1). Rice (*Oryza sativa* L.) cultivar Nipponbare was grown in an experimental field at Tohoku University (Kashimadai, Miyagi, Japan) in 2009, and bacterial DNA was extracted from the roots using the bacterial cell enrichment method (6).

Using the 454 read sample, 16S rRNA genes were amplified with the primer set, Bac-27F (5′-CCTATCCC CTGTGTGCCTTGGCAGTCTCAG_agagtttgatcmtggctca-3′), MID-518R (5′-CCATCTCATCCCTGCGTGTCTCCGA CTCAG_ barcode (MID)_ ttaccgcggctgctgg-3′), where nucleotide sequences shown in lowercase letters are universal sequences of bacterial 16S rRNA genes. MID-518R primer contains the sequences of the Titanium A adaptor, Key sequence (TCAG) and barcode sequences (MIDs), while Bac-27F primer contains the sequences of the Titanium B adaptor and Key sequence (TCAG). These PCR primers target the V1–V3 regions (1). Three independent samples were sequenced on a 454 GS-FLX pyrosequencer with three barcodes (MIDs). MID sequences used as samples 1, 2 and 3 in this study were MID1 (5′-ACGAGTGCGT), MID2 (5′-ACGCTCGACA) and MID3 (5′-AGACGCACTC), respectively.

As for 454 reads, sequences were assigned to each sample according to sample-specific barcodes, and were used to simulate the effect of read length on the taxonomic assignment of 16S rRNA gene reads. Regions corresponding to the first 250, 300, 350, and 400 bases of the 518R primer were independently retrieved from the original sequences, and were designated P518R-250, -300, -350, and -400 (Fig. 1, Table 2). Sequences shorter than the aimed-at length were removed. Low-quality sequences were then eliminated with the RDP Pyrosequencing Pipeline for a maximum edit distance of primer 518R=0, average quality score ≥25, and the maximum number of ambiguous characters (denoted by N)=0. The regions between primers 518R and 27F (P518R-27F sample) and between primers 518R and 109F (P518R-109F sample) were also retrieved from the original sequences (Fig. 1) with the RDP Pyrosequencing Pipeline for a maximum edit distance of primer 518R=0, a maximum edit distance of primer 27F or 109F=2, average quality score ≥25, and the maximum number of ambiguous characters (N)=0. Potentially chimeric sequences were removed by Chimera Slayer (4, 16) with default parameters. The remaining sequences were used in independent phylogenetic analyses. Taxonomic assignment for each simulated sample was conducted using the RDP MultiClassifier tool with a minimum support threshold of 80% or 50% (19).

In Sanger reads, 16S rRNA genes were amplified with the universal primers 27F (5′-AGAGTTTGATCMTGGCTCAG-3′) and 1525R (5′-AAGGAGGTGWTCCARCC-3′), and sequences were analyzed on a Type 3730xl DNA Analyzer (Applied Biosystems, Foster City, CA, USA) using the 27F primer. As for the Sanger reads, sequences that did not contain a perfect match with the 518R primer sequence were removed. The remaining sequences designated S665R-109F (corresponding to 109–665 bp of *Escherichia coli* 16S rRNA gene) were used to simulate the effect of read length on the taxonomic assignment of 16S rRNA gene reads. Regions corresponding to the first 250, 300, and 350 bases of the 518R primer were independently retrieved from the region between the 518R and 109F primers, and were designated S518R-250, -300, and -350 samples (Fig. 1). The region between primers 518R and 109F (S518R-109F) was retrieved from the S665R-109F sample with the RDP Pyrosequencing Pipeline tool (http://pyro.cme.msu.edu/init/form.spr) for maximum edit distances of primer 518R=0 and of primer 109F (5′-ACGGGTGM-GTAACRCGT-3′)=2. Potentially chimeric sequences were removed with Chimera Slayer software (4, 16) with default parameters. The remaining sequences were used in independent phylogenetic analyses.

Among the Sanger samples analyzed using the RDP MultiClassifier tool with a minimum support threshold of 80%, the phylogenetic compositions of all simulated samples were almost identical to that of the S665R-109F sample down to family level (Figs. 2A and 2C). Those of the S518R-109F and S518R-350 samples were almost identical to that of the S665R-109F sample at the genus level (Fig. 2E), except that *Methylocystis* was not detected in the former two; however, the relative abundances of *Bradyrhizobium* and *Methylosinus* in the S518R-300 and -250 samples were much lower than in the other Sanger samples (Fig. 2E). Among the 454 samples also, the relative abundances of *Bradyrhizobium* and *Methylosinus* in P518R-300 and -250 were much lower than those in the other 454 samples (Fig. 2F). These results suggest that most of the *Bradyrhizobium* and *Methylosinus* reads could not be classified accurately by using the region corresponding to the first 250 to 300 bases of the 518R primer.

Among the 454 samples analyzed using the RDP Multi-Classifier tool with a minimum support threshold of 80%, P518R-400, P518R-109F, P518R-350, and P518R-300 and P518R-250 showed almost identical phylogenetic compositions down to family level (Fig. 2B, 2D, 2F) On the other hand, P518R-27F showed a higher abundance of *Alphaproteobacteria* than the other 454 samples (Fig. 2B), which was due mostly to the high abundance of *Bradyrhizobiaceae* and *Methylocystaceae* (Fig. 2D). Interestingly, the relative abundance of *Bradyrhizobium* was much higher in P518R-27F than that in the other 454 samples (Fig. 2F), suggesting that some *Bradyrhizobium* reads could not be classified accurately by using the region corresponding to the first 250 to 400 bases of the 518R primer. To assess the read length effect in the taxonomic assignment of *Bradyrhizobium* reads, we retrieved the regions corresponding to the first 250, 300, 350, and 400 bases of the 518R primer from the reads assigned as *Bradyrhizobium* in the P518R-27F sample and analyzed them independently. Although more than 99% of the reads were correctly classified to family level in all simulated data sets, the accuracy of phylogenetic assignment at genus level depended largely on read length (Fig. 3). These results suggest that the P518R-400 and shorter samples are inappropriate for surveying the relative abundance of *Bradyrhizobium*. Although the region between 518R and 27F would be practical for surveying the relative abundance of *Bradyrhizobium* and *Methylosinus*, the relative abundance of the clostridia (*Firmicutes*) was much lower in P518R-27F than in the other 454 samples, suggesting that the region between primers 518R and 27F was not suitable for detecting *Firmicutes*.

To examine the effects of a lower threshold value on the taxonomic assignment using RDP MultiClassifier, both 454 and Sanger samples were also analyzed with a minimum support threshold of 50% (Figs. 2G–2H, S1). Similar assignments to genera were observed except for the 300 bp length (S518R-300 and P518R-300) between the two different thresholds (80% and 50%). In particular, the relative abundance of *Bradyrhizobium* was extremely low at 250 bp (S518R-250 and P518R-250) as compared with other lengths of sequences (Figs. 2, 3, S2).

Although RDP MultiClassifier is a very useful tool, the accuracy of assignment is somewhat diminished in analyses of short length reads (10). Thus, we examined whether the BLASTN-based approach might improve phylogenetic assignments. First, to build a BLASTN database, SILVA SSU Ref NR Release 108 was downloaded from the Silva web site (http://www.arb-silva.de/). Sequences assigned as environmental samples (taxonomy ID, 48479) and unclassified sequences (taxonomy ID, 12908) were removed from the downloaded file. Remaining sequences were used as a BLASTN database. Taxonomic assignment for each simulated sample was conducted according to best-hit pairs in BLASTN analysis (NCBI’s blastall version 2.2.24) against the in-house SILVA SSU Ref NR database. BLASTN results were filtered by e-value (≤1.0e-30), hit length coverage (≥90% of a query sequence) and similarity (≥90%). The taxonomic assignment by BLASTN analysis (Fig. 2I–2J, Table S1) was compared with that of RDP MultiClassifier analysis (Fig. 2E–2H). Although the BLASTN-based approach was able to detect *Methylosinus* even in 250-bp sequences (S518R-250 and P518R-250), no assignment to *Bradyrhizobium* was observed using 250-bp Sanger and 454 sequences (Fig. 2E–2H, S518R-250 and P518R-250).

To assess the difference caused by using different sequencers and primer sets, we compared the phylogenetic compositions of the 454 and Sanger samples (Fig. 2, Table S1). The 454 samples showed a higher abundance of *Alphaproteobacteria* and a lower abundance of *Betaproteobacteria* than in all corresponding Sanger samples. So far we have been unable to explain what caused these differences. Because independent DNA samples were amplified using different PCR primer sets and sequenced on different sequencers, it is relevant that high abundances of *Bradyrhizobium*, *Burkholderia*, and *Methylosinus* were observed in both 454 and Sanger samples, and the magnitude relation among those genera was roughly consistent between Sanger and 454 samples.

The accuracy of phylogenetic assignment at genus level depends largely on read length, especially in some genera such as *Bradyrhizobium* and *Methylosinus*. The members of *Bradyrhizobium* are ecologically important nitrogen-fixing bacteria (8, 11, 12, 15, 17, 18). *Methylosinus* is a representative methane oxidizer in paddy fields and plays important roles in the methane cycle there (12). In 16S rRNA sequence analysis, sequences shorter than 400 bases might cause erroneous phylogenetic assignment in rice root microbiomes; therefore, it is reasonable to remove sequences shorter than 400 bases for phylogenetic analysis at genus level. The region between primers 518R and 27F would be suitable for surveying the abundance of *Bradyrhizobium* and *Methylosinus*; however, the relative abundance of *Firmicutes* was much lower than in the other 454 samples. These results suggest that the target region of 16S rRNA gene sequences should be selected for the purposes of each study (2, 9).

The phylogenetic composition of the 454 samples was analyzed using three independent DNA samples extracted from rice grown in the same field. The phylogenetic stability of each 454 sample was assessed (Table 3). At phylum level, the compositions were almost identical within three replicates; however, at genus level, sample 3 showed an apparently different composition of *Burkholderia* (29%) from the other two samples (17–20%) (Table 3). On the other hand, the relative abundance of *Bradyrhizobium* remained stable (28–33%). Three independent analyses of 454 samples showed how the perceived phylogenetic diversity can be easily influenced by experimental procedures, emphasizing the need for multiple independent analyses to reduce bias (Table 3). It is important to carefully examine the effects of several variables on community composition estimates, such as biases due to bioinformatic analysis, sample preparation (6), DNA extraction, or PCR conditions (3) for respective microbiomes.

## Supplementary material



## Figures and Tables

**Fig. 1:**
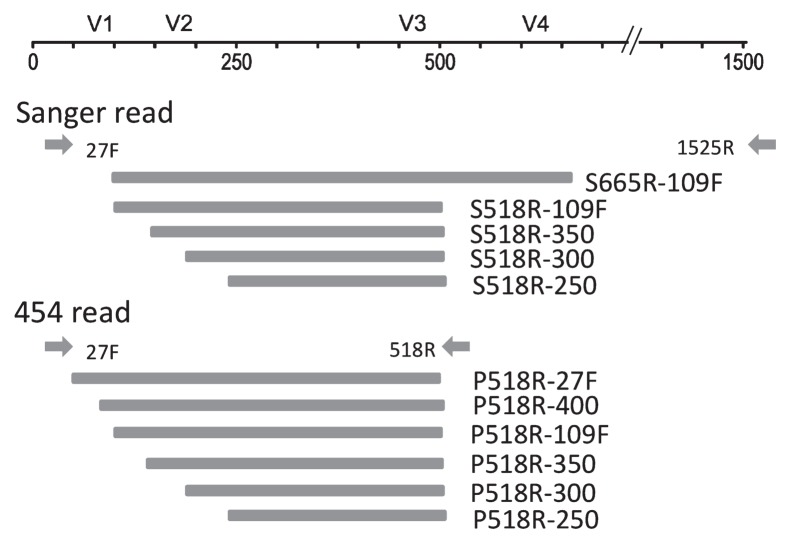
Positions of primer sequences (arrows) and 16S rRNA gene regions (bars) used for Sanger and 454 reads.

**Fig. 2:**
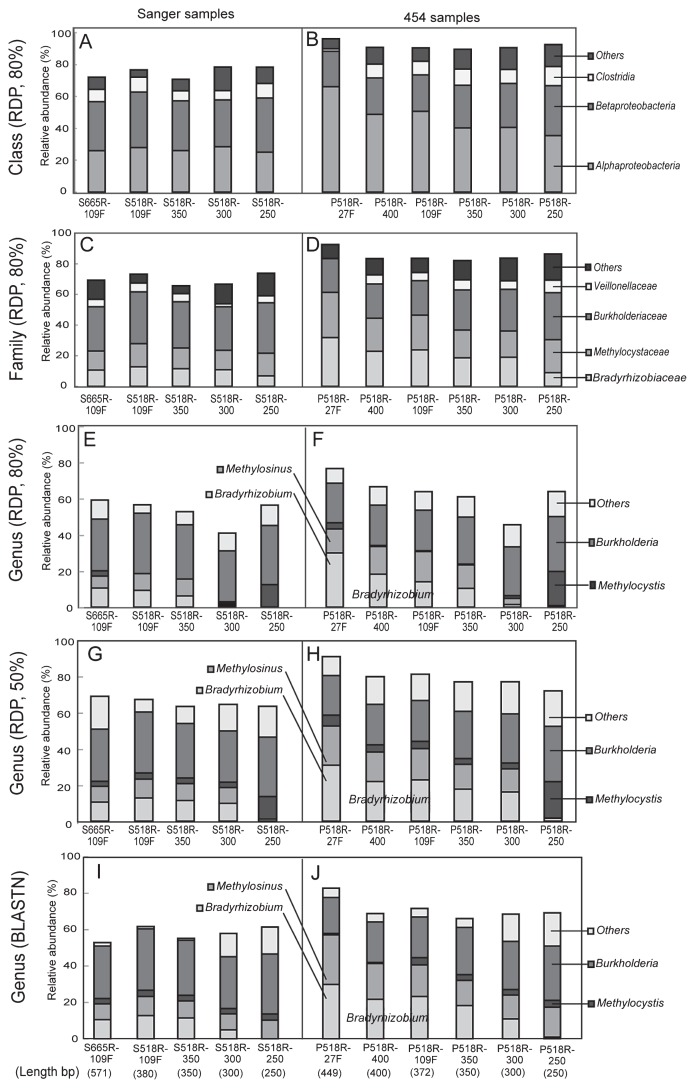
Phylogenetic compositions of 16S rRNA libraries of Sanger sample (S665R-109F), 454 sample (P518R-27F) and simulated samples with different lengths retrieved from the original samples. A, C, E, F, G and I: Profiles of Sanger samples at class, family, and genus levels. B, D, F, H and J: Profiles of 454 samples at class, family, and genus levels (means of three independent analyses). Taxonomic assignment was conducted using RDP MultipleClassifier with a minimum support threshold of 80% (A–F) and 50% (G–H), and using BLASTN with hit length coverage ≥90% and similarity ≥90% (I–J). Original data of A–F and I–J are shown in Table S1.

**Fig. 3:**
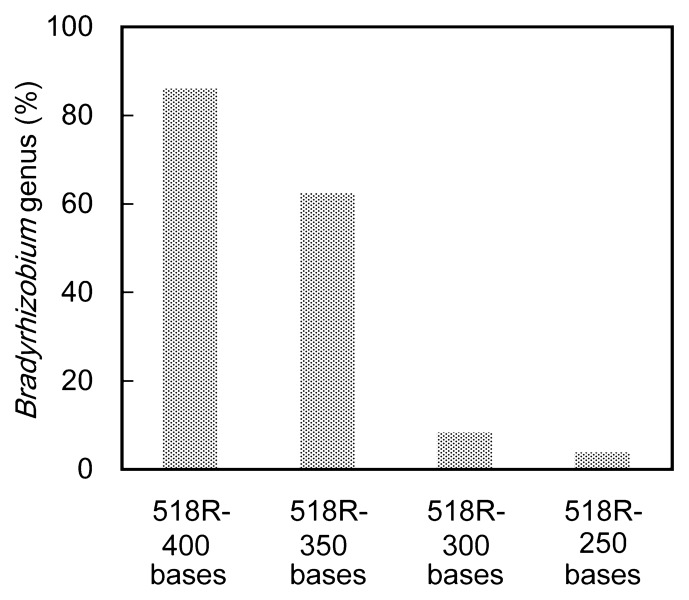
Proportions of reads assigned as *Bradyrhizobium* by use of partial *Bradyrhizobium* sequences with different lengths retrieved from *Bradyrhizobium* reads in P518R-27F sample. Taxonomic assignment was conducted using RDP MultipleClassifie with a minimum support threshold of 80%.

**Table 1:** Sequence data sets used^a^

	No. of reads	PCR amplification primer		Sequencing primer	Sequencing method	Accession numbers

		Forward	Reverse			
Sanger reads	112	27F	1525R	27F	Sanger dideoxy sequencing	AB579660–765
454 reads 1	6,237	27F	518R	518R	Pyrosequencing (454)	DRS000517
454 reads 2	5,333	27F	518R	518R	Pyrosequencing (454)	DRS000518
454 reads 3	50,266	27F	518R	518R	Pyrosequencing (454)	DRS000519

a The project number for 454 reads in the NCBI database is ID 61421.

**Table 2:** Summary of original and simulated samples

	No. of reads	Length (bases)
Sanger read		
S665R-109F	104	571^a^
S518R-109F	86	380^a^
S518R-350	96	350
S518R-300	102	300
S518R-250	88	250

454 read		
P518R-27F		
454 reads 1	1,824	447^a^
454 reads 2	1,438	448^a^
454 reads 3	13,380	452^a^
P518R-109F		
454 reads 1	2,937	370
454 reads 2	2,411	370
454 reads 3	22,761	370
P518R-400		
454 reads 1	3,013	400
454 reads 2	2,499	400
454 reads 3	23,078	400
P518R-350		
454 reads 1	4,311	350
454 reads 2	3,735	350
454 reads 3	35,735	350
P518R-300		
454 reads 1	4,066	300
454 reads 2	3,576	300
454 reads 3	34,177	300
P518R-250		
454 reads 1	3,671	250
454 reads 2	3,399	250
454 reads 3	32,955	250

a Average length of all reads in the sample.

**Table 3:** Phylogenetic composition of three independent P518R-27F samples of 454 reads

	Relative abundance (%)		
	
	Sample 1	Sample 2	Sample 3
Phylum			
*Proteobacteria*	88.8	89.3	91.9
Others	8.1	7.2	5.5
Class			
*Alphaproteobacteria*	69.5	67.7	61.2
*Betaproteobacteria*	17.4	20.0	29.3
Others	9.2	8.3	6.1
Order			
*Rhizobiales*	66.9	64.7	57.8
*Burkholderiales*	17.2	19.9	29.3
Others	9.1	8.3	6.3
Family			
*Bradyrhizobiaceae*	34.8	29.3	31.3
*Methylocystaceae*	29.9	33.7	25.1
*Burkholderiaceae*	17.0	19.9	29.2
Others	11.0	9.2	7.5
Genus			
*Bradyrhizobium*	32.7	27.8	29.8
*Methylosinus*	11.9	15.3	13.1
*Methylocystis*	3.5	3.9	3.0
*Burkholderia*	17.0	19.9	29.2
Others	10.6	7.9	6.2

Underlines indicate taxon with markedly different relative abundances among samples.
