# Supplementary material for: Pyrosequence Read Length of 16S rRNA Gene Affects Phylogenetic Assignment of Plant-associated Bacteria
Source: Microbes Environ. 2012 Feb 22;27(2):204–8. doi: 10.1264/jsme2.ME11258 (PMC4036018; doi:10.1264/jsme2.ME11258)
Supplement: Supplementary file 1 [file 27_204_s1.pdf]

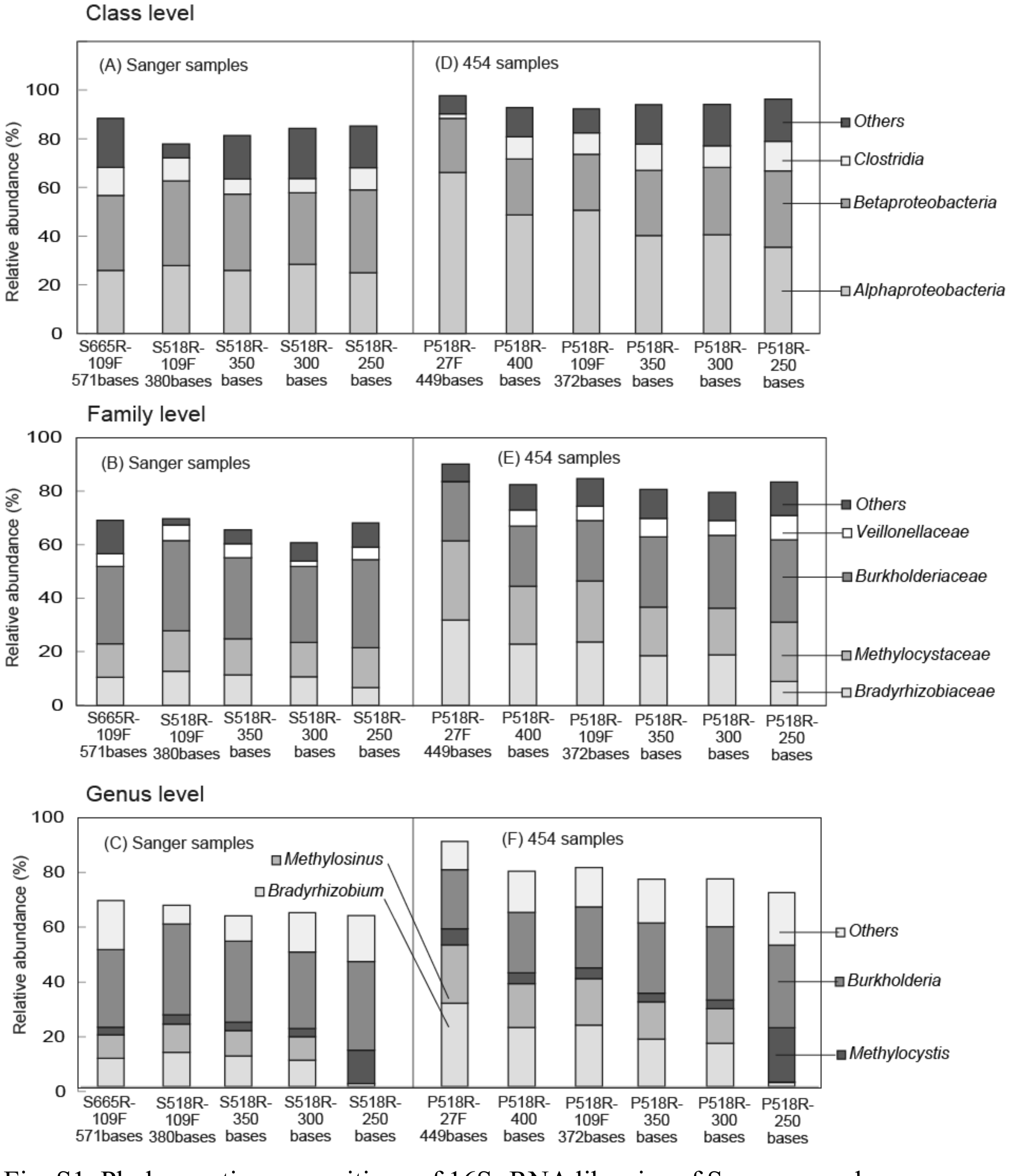

Fig. S1. Phylogenetic compositions of 16S rRNA libraries of Sanger sample (S665R-109F), 454 sample (P518R-27F) and simulated samples with different lengths retrieved from the original samples. (A–C) Profiles of Sanger samples at class, family, and genus levels. (D–F) Profiles of 454 samples at class, family, and genus levels (means of three independent analyses). Taxonomic assignment was conducted using RDP MultipleClassifier with with a minimum support threshold of 50%.

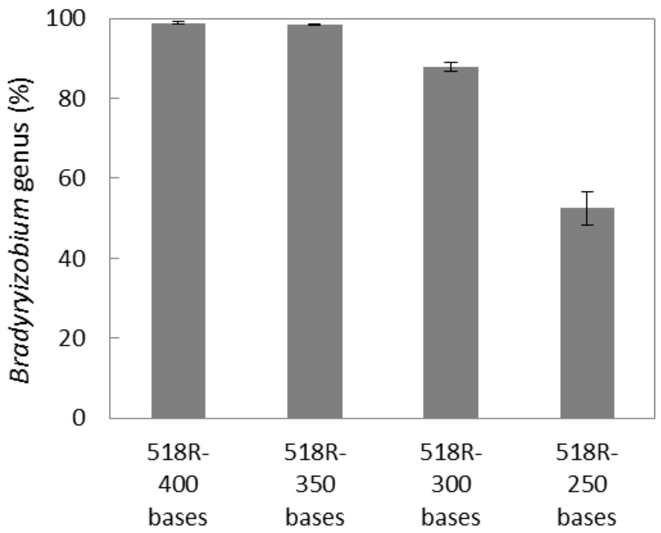

Fig. S2. Proportions of reads assigned as *Bradyrhizobium* by use of partial *Bradyrhizobium* sequences with different lengths retrieved from *Bradyrhizobium* reads in P518R-27F sample. Taxonomic assignment was conducted using RDP MultipleClassifier with a minimum support threshold of 50%. Graph shows average ± standard error

Table S1. Phylogenetic compositions of 16S rRNA libraries of Sanger read sample (S665R-109F), 454 read sample (P518R-27F), and simulated samples with different lengths retrieved from the original samples.
Relative abundance of 454 samples shows the average of three independent analyses.

	Phylogenetic composition (%)																					
	RDP MultipleClassifier [a]											BLASTN										
	Sanger sample					454 sample						Sanger sample					454 sample					
	S665R-109F (571 bases)	S518R-109F (380 bases)	S518R-350 (350 bases)	S518R-300 (300 bases)	S518R-250 (250 bases)	P518R-27F (449 bases)	P518R-400 (400 bases)	P518R-109F (372 bases)	P518R-350 (350 bases)	P518R-300 (300 bases)	P518R-250 (250 bases)	S665R-109F (571 bases)	S518R-109F (380 bases)	S518R-350 (350 bases)	S518R-300 (300 bases)	S518R-250 (250 bases)	P518R-27F (449 bases)	P518R-400 (400 bases)	P518R-109F (372 bases)	P518R-350 (350 bases)	P518R-300 (300 bases)	P518R-250 (250 bases)
Phylum																						
Proteobacteria	65.4	66.3	64.6	72.5	69.3	90.0	76.0	77.6	73.5	76.8	74.3	55.8	62.8	57.3	60.8	60.2	87.7	72.2	74.3	68.6	70.8	69.7
Firmicutes	7.7	9.3	7.3	6.9	9.1	1.6	9.5	9.5	11.2	9.7	12.2	1.0	1.2	0.0	2.9	4.5	0.3	0.9	0.9	1.3	2.2	3.8
Cyanobacteria	0.0	0.0	0.0	0.0	0.0	0.2	0.1	0.2	0.2	0.2	0.2	6.7	9.3	8.3	11.8	12.5	0.9	2.2	2.4	2.7	3.8	3.4
Others	2.9	1.2	3.1	2.9	3.4	3.2	5.9	4.1	6.9	6.5	7.4	1.9	1.2	2.1	2.0	3.4	1.9	2.0	1.2	2.6	3.5	5.2
Class																						
Alphaproteobacteria	26.0	27.9	26.0	28.4	25.0	66.1	48.7	50.7	40.2	40.6	35.5	24.0	27.9	25.0	28.4	25.0	65.1	46.7	49.0	38.7	40.4	35.4
Betaproteobacteria	30.8	34.9	31.3	29.4	34.1	22.2	23.0	22.9	26.8	27.6	31.4	30.8	34.9	31.3	29.4	34.1	22.2	22.9	22.9	26.7	27.5	31.4
Clostridia	7.7	9.3	6.3	5.9	9.1	1.6	8.6	8.5	10.2	8.8	12.0	1.0	1.2	0.0	2.0	3.4	0.2	0.5	0.6	0.8	1.3	1.3
Others	7.7	4.7	7.3	14.7	10.2	6.3	10.5	8.5	12.4	13.8	13.8	1.9	0.0	2.1	2.9	3.4	2.2	3.3	2.2	4.5	6.3	8.8
Order																						
Rhizobiales	24.0	27.9	25.0	26.5	23.9	63.2	46.2	48.5	38.3	38.9	33.9	24.0	27.9	25.0	25.5	21.6	61.3	44.7	47.6	37.5	38.7	32.1
Burkholderiales	28.8	33.7	30.2	28.4	33.0	22.1	22.7	22.6	26.5	27.5	30.9	28.8	33.7	30.2	28.4	33.0	19.8	22.5	22.4	26.2	26.7	30.1
Clostridiales	7.7	9.3	5.2	5.9	9.1	1.6	8.6	8.5	10.2	8.7	12.0	1.0	1.2	0.0	2.0	3.4	0.2	0.5	0.6	0.8	1.3	1.3
Others	8.7	2.3	6.3	7.8	9.1	6.3	8.2	5.8	9.4	9.9	12.3	1.0	0.0	1.0	2.9	5.7	2.6	3.2	2.1	3.8	5.7	8.8
Family																						
Bradyrhizobiaceae	10.6	12.8	11.5	10.8	6.8	31.8	22.9	23.7	18.6	19.0	8.9	10.6	12.8	11.5	10.8	6.8	31.2	22.7	23.7	18.6	18.9	8.6
Methylocystaceae	12.5	15.1	13.5	12.7	14.8	29.5	21.6	22.7	18.1	17.2	21.5	12.5	15.1	13.5	12.7	14.8	28.8	20.9	22.0	17.5	16.7	20.8
Burkholderiaceae	28.8	33.7	30.2	28.4	33.0	22.0	22.5	22.6	26.2	27.2	30.7	28.8	33.7	30.2	28.4	33.0	19.8	22.3	22.3	26.0	26.5	29.9
Veillonellaceae	4.8	5.8	5.2	2.0	4.5	0.1	5.9	5.3	6.6	5.6	8.2	0.0	0.0	0.0	0.0	0.0	0.0	0.1	0.1	0.3	0.5	1.4
Others	12.5	5.8	5.2	12.7	14.8	9.1	10.7	9.3	12.6	14.9	17.1	2.9	1.2	1.0	5.9	9.1	3.2	3.8	4.2	4.3	7.8	9.0
Genus																						
Bradyrhizobium	10.6	9.3	6.3	1.0	0.0	30.1	18.3	14.1	10.4	1.4	0.8	10.6	12.8	11.5	4.9	0.0	29.9	21.7	23.3	18.3	10.9	1.0
Methylosinus	6.7	9.3	9.4	1.0	0.0	13.4	15.5	16.8	13.2	3.5	0.0	8.7	10.5	9.4	8.8	10.2	27.2	19.7	17.3	13.8	13.1	16.4
Methylocystis	2.9	0.0	0.0	1.0	12.5	3.4	0.6	0.5	0.4	1.5	19.0	2.9	3.5	3.1	2.9	3.4	0.7	0.6	4.0	3.1	3.0	3.8
Burkholderia	28.8	33.7	30.2	28.4	33.0	22.0	22.5	22.6	26.2	27.2	30.6	28.8	33.7	30.2	28.4	33.0	19.8	22.3	22.3	26.0	26.5	29.9
Others	10.6	4.7	7.3	9.8	11.4	8.2	10.2	10.3	11.3	12.3	13.9	1.9	1.2	1.0	12.7	14.8	5.1	4.6	4.7	4.8	15.0	18.2

[a] Taxonomic assignment was conducted using RDP MultipleClassifier with a minimum support threshold of 80%.
